# Necrotizing Soft Tissue Infections of the Breast: A Potentially Lethal Surgical Emergency

**DOI:** 10.1155/2023/4695019

**Published:** 2023-07-20

**Authors:** Shariful Islam, Aneela Shah, Anthony Maughn, Sarah Dial, Avidesh Mahabir, Vijay Naraynsingh, Patrick Harnarayan

**Affiliations:** ^1^Department of General Surgery, Breast Unit, San Fernando General Hospital, San Fernando, Trinidad and Tobago; ^2^Department of Clinical Surgical Sciences, University of the West Indies, St. Augustine, Trinidad and Tobago; ^3^Medical Associates Hospital, St. Joseph, Trinidad and Tobago

## Abstract

Necrotizing soft tissue infection (NSTI) of the breast is an extremely rare event in surgical practice. It is considered the most aggressive form of soft tissue infection and a true surgical emergency. It is also associated with a high risk of mortality if not diagnosed promptly. Few cases have been documented in the literature; the exact etiology and risk factors vary from those involving the limbs, trunk, and perineum. Early recognition, prompt surgical treatment, and broad-spectrum antibiotic therapy are crucial for reducing morbidity and mortality. These reports present cases of NSTIs in breasts with unique etiologies and challenges in their management.

## 1. Introduction

Necrotizing fasciitis of the breast is a clinical entity infrequently described in surgical texts and rarely encountered in practice. Unlike other necrotizing soft tissue infections (NSTIs), this entity occurs due to a variety of causes, including local trauma, breast infarction, and topical applications. Mortality rates have been reported as high as 73% but can be reduced with early diagnosis and the rapid initiation of appropriate multidisciplinary strategies [1].

We present four cases of breast NSTI, each with unique etiologies and challenges along with their management pathway. We seek to discuss the incidence of this rare pathology, its associated microbiology, and the options for surgical management.

## 2. Case 1

**Figure 1:**
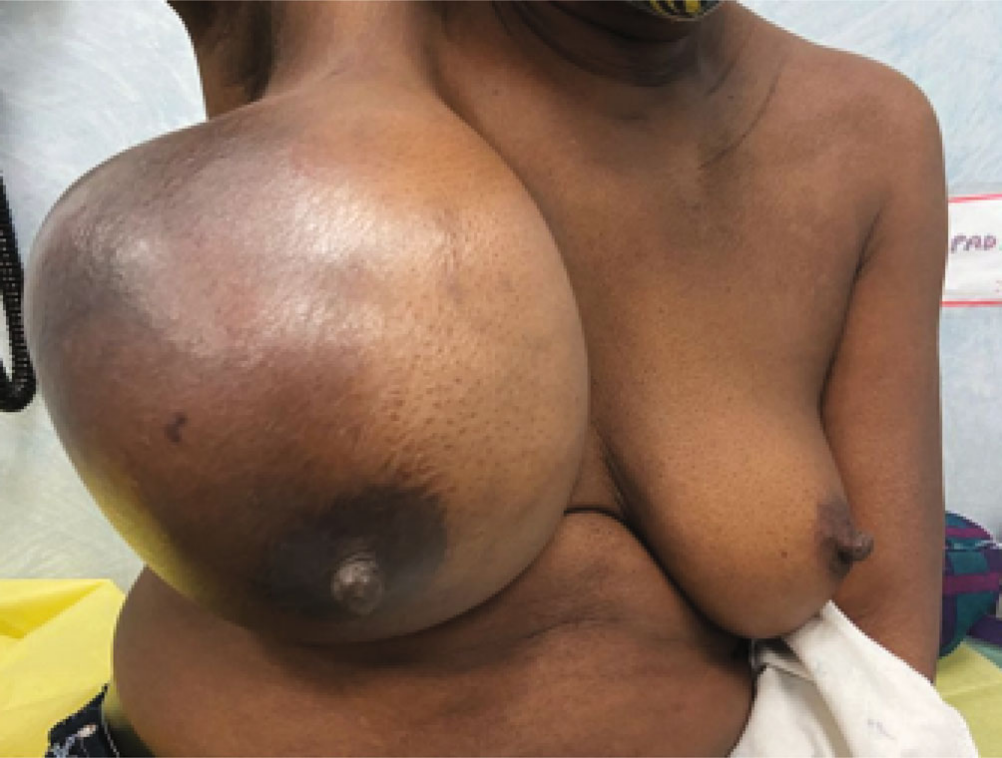
Image showing the appearance of the right breast two months before presentation.

A 60-year-old female presented to the Accident and Emergency Department with a “ruptured right breast.” She was diagnosed approximately ten months prior with triple negative, locally advanced (T3N2M0) breast carcinoma, which progressed to T4bN2M0 disease while on neoadjuvant chemotherapy (Figure 1). She was reviewed two months before the presentation, with a decision to proceed with a modified radical mastectomy. However, she declined further treatment.

**Figure 2:**
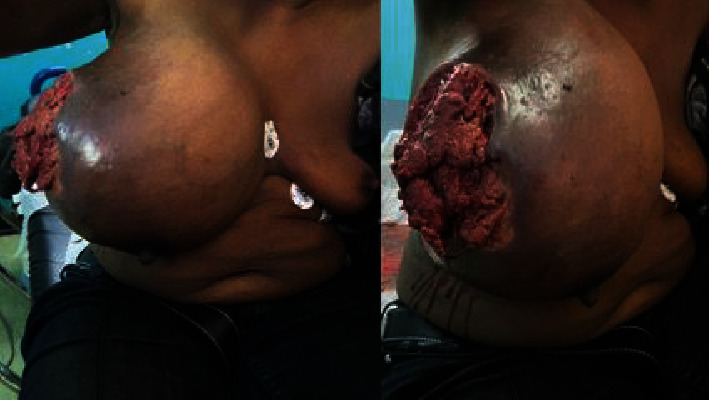
Image showing a large ulcerated tumor at acute presentation.

On assessment, the patient was hypotensive (blood pressure 94/69 mmHg), tachycardic with a pulse of 140 beats per minute, respiratory rate of 34 breaths per minute, and oxygen saturation of 98% in room air. There was a large ulceration of the tumor at the lateral aspect of the right breast with a foul odor and significant drainage of turbid fluid (Figure 2). The right breast was warm, with gas bubbles noted at the wound, and axillary lymphadenopathy. No abnormalities in the left breast were identified.

**Figure 3:**
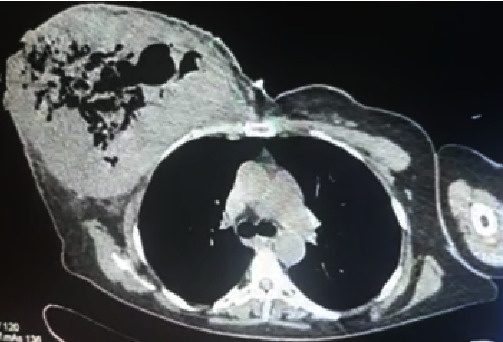
CT scan of the chest showing gas within the right breast and tumor invasion into the right pectoral muscles.

Laboratory investigations revealed normocytic anemia [hemoglobin (Hb) 5.5 g/dL], leukocytosis (29 × 10^9^/L), hyponatremia (129 mmol/L), hypokalemia (3.3 mmol/L), acute kidney injury, and serum creatinine 2.2 mg/dL. Non-contrast computerized tomography (CT) showed a significant amount of gas throughout the right breast, with an invasion of the tumor into the pectoral muscles (Figure 3). The patient was resuscitated with intravenous fluids and given piperacillin–tazobactam. She underwent an emergency right radical mastectomy with delayed primary closure.

**Figure 4:**
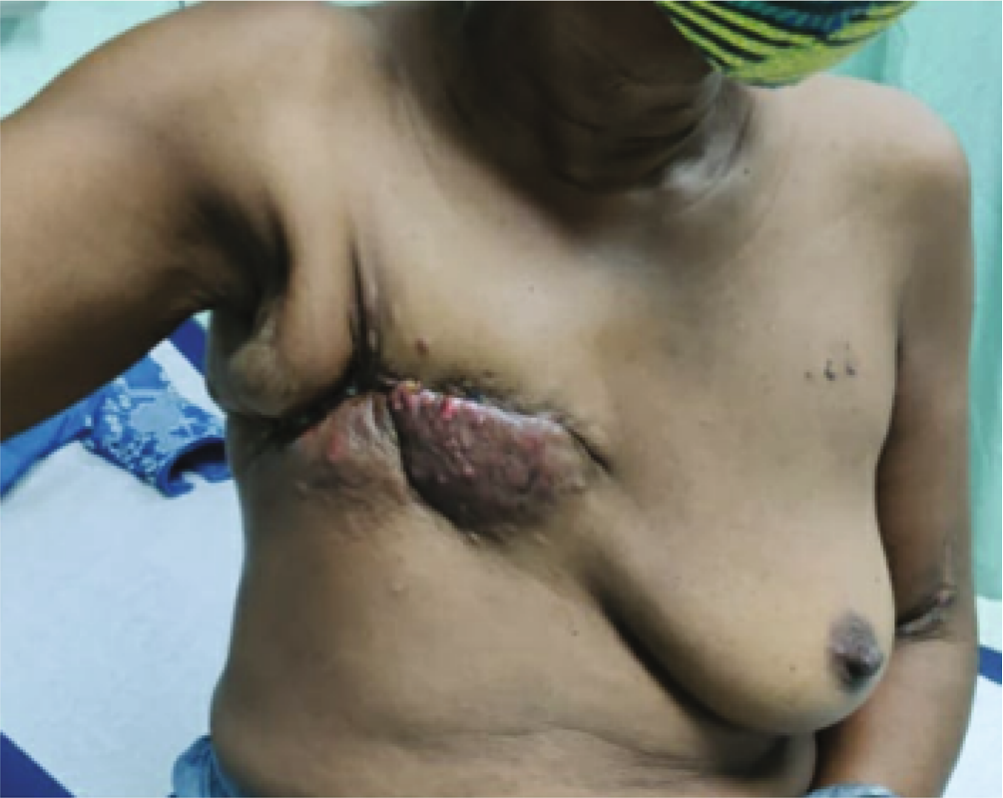
Image showing recurrent disease on the right chest wall.

The single causative organism based on tissue culture was *Escherichia coli*. She had an uneventful postoperative recovery and was discharged one week later. However, she was readmitted on day 12 with dyspnea and tachycardia and was treated for bilateral pulmonary emboli. She subsequently defaulted from follow-up for adjuvant therapy and re-presented six months later with a recurrence at the chest wall, confirmed histologically (Figure 4). She died three weeks later when she developed acute confusion due to multiple brain metastases.

## 3. Case 2

A 60-year-old diabetic and hypertensive female was referred from the Accident and Emergency Department with a two-week history of pain, redness, and swelling of the left breast. The working diagnosis at referral was a breast abscess. She reported having a sebaceous cyst that became infected and worsened despite oral antibiotics and topical remedies (soft candles).

**Figure 5:**
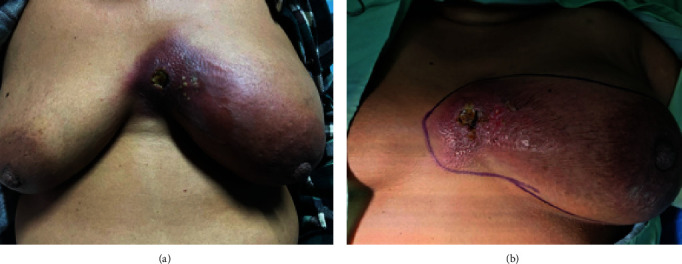
Image showing necrotizing skin and soft tissue infections of the left breast. (a) Image of the both breast of the patients showing necrotizing infection involving medial side of the left breast. (b) A closer view of the left breast showing ulceration and infected areas of the left breast (see the marking).

At the examination, the patient appeared unwell, with a blood pressure of 133/56 mmHg, a pulse of 133 beats per minute, a respiratory rate of 30 breaths per minute, and an oxygen saturation of 99% in room air. There was extensive erythema in the medial aspect of the left breast, involving the nipple–areola complex and skin necrosis at the site of the sebaceous cyst (Figure 5). There was palpable crepitus throughout the breast, with purulent discharge. No axillary lymphadenopathy was noted.

**Figure 6:**
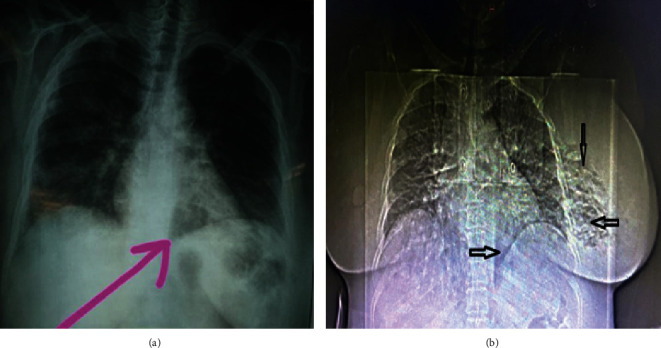
Chest X-ray posteroanterior view demonstrating suspicion of subcutaneous gas (see arrows).

**Figure 7:**
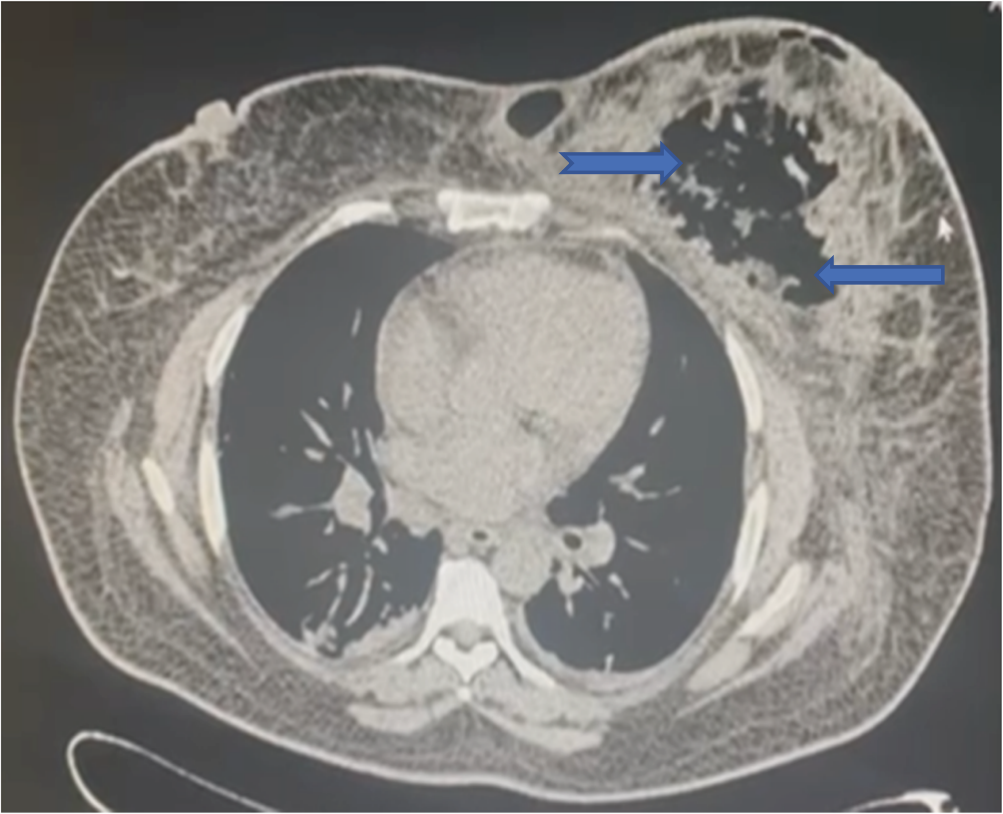
CT scan of the chest showing extensive gas within the left breast (see blue arrows).

Blood investigations revealed leukocytosis (33 × 10^9^/L) and acute kidney injury (serum creatinine 1.6 mg/dL). A chest X-ray appeared suspicious for subcutaneous emphysema of the left chest wall (Figure 6), and CT confirmed extensive gas within the left breast (Figure 7).

**Figure 8:**
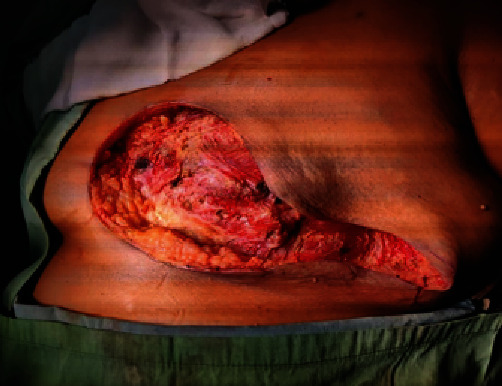
Image of the open wound after an emergency left mastectomy.

An emergency left mastectomy was performed, and the wound was left open for delayed closure (Figure 8). The patient had an uncomplicated recovery, with debridement of the superior skin flap edge forty-eight hours later. Tissue cultures grew *Klebsiella* and *Enterobacter.* Our patient refused any flap reconstruction; hence, the wound was closed by local flap advancement after 4 weeks.

## 4. Case 3

A 32-year-old obese female with known anal cancer and bilateral pulmonary embolism (PE) presented to the Accident and Emergency Department with one week of history of pain, swelling, redness, and discharge of pus from her left breast. There was no history of trauma or any insect bites on her breast, and she complained of a mild fever for 48 hours. She completed her second cycle of chemotherapy and was on rivaroxaban for her bilateral PE.

**Figure 9:**
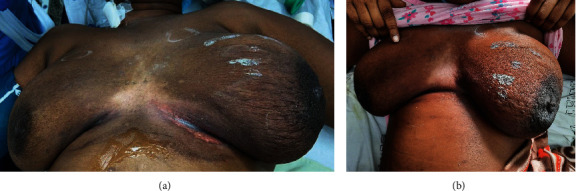
Image of the left breast showing the swelling, redness, and discharging sinus (see arrows). (a) Image of both breast showing that left breast is swollen and larger than the right breast. (b) Closer view of the left breast showing redness of skin of the left breast with a discharging sinus just medial to the left nipple.

On examination, the patient appeared unwell, with a blood pressure of 142/77 mmHg, a pulse of 128 beats per minute, a respiratory rate of 26 breaths per minute, and an oxygen saturation of 95% in room air. There was mild erythema of the lower inner quadrant of the left breast with a 10 cm × 10 cm tender mass, no palpable crepitus but purulent discharge (Figure 9), and there was no palpable axillary lymphadenopathy.

**Figure 10:**
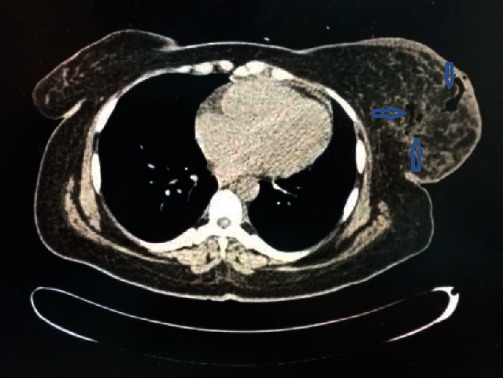
CT scan of the chest showing gas within the left breast (see blue arrows).

Lab investigations revealed leukocytosis (27.6 × 10^9^/L), Hb of 7.2 gm/dL, and normal platelet and kidney function tests. A CT scan of the chest confirmed extensive gas within the left breast (Figure 10).

**Figure 11:**
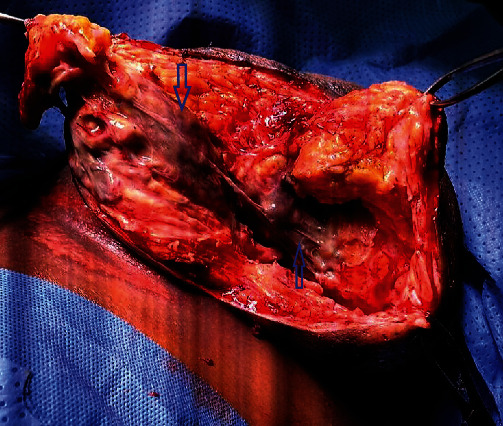
Intra-operative image showing the necrotic fascia of the left breast (see blue arrows).

**Figure 12:**
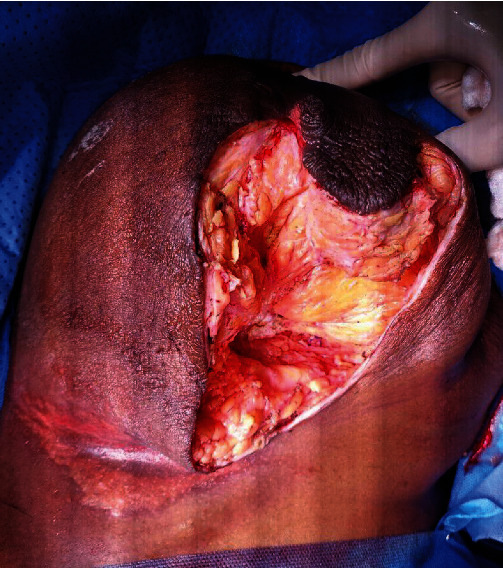
Post-debridement image showing a clean wound on the left breast (left open).

The patient consented to wide debridement of the left breast. The infected tissue and necrotic fascia were completely excised (Figure 11). The wound was left open with a plan for secondary closure once all infections subside (Figure 12). The culture grew *E. coli* and *Klebsiella*. Unfortunately, our patient died after a few weeks of PE.

## 5. Case 4

A 36-year-old female patient presented to the Emergency Department with a five-day history of pain, swelling, and redness in her right breast and a one-day history of fever. The patient gave a history of having had an insect bite in her right breast seven days ago.

**Figure 13:**
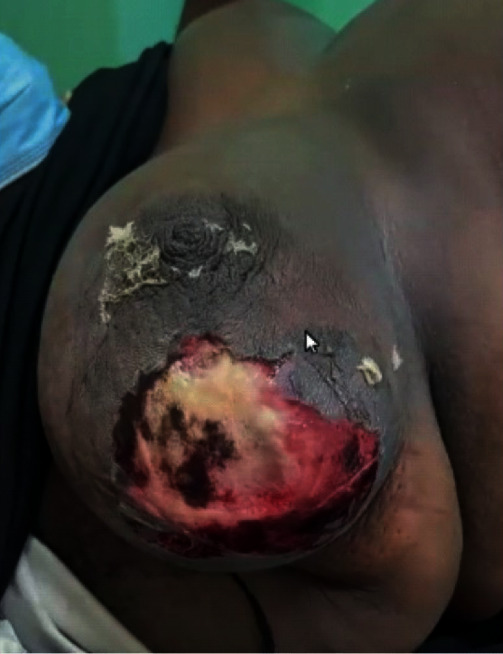
Image of the right breast showing desquamation with necrotic patches over the breast.

On examination in the Emergency Department, the patient was obese, ill-looking, tachycardic with a pulse of 120 beats per minute, hypotensive with a blood pressure of 96/58 mmHg, respiratory rate of 25 breaths per minute, and oxygen saturation of 96% on room air. The entire right breast was swollen and edematous, and the outer half of her right breast was red with a 10 cm × 10 cm area of desquamation with areas of necrotic underlying tissue (Figure 13). The right breast was warm and tender to touch, and there was no palpable crepitus or axillary lymphadenopathy.

Her laboratory investigation revealed marked leukocytosis (26.26 × 10^9^/L), Hb of 10 g/dL, a normal platelet count, and a kidney function test.

The patient was resuscitated, a broad-spectrum antibiotic was started, and she consented to emergency-wide debridement of the right breast. The wound was left open, and delayed primary closure of the right breast with contralateral reduction mastopexy was done to achieve symmetry. The tissue culture grew *E. coli*.

## 6. Discussion

Necrotizing fasciitis is a life-threatening infection that involves deep soft tissue layers (muscle fascia and overlying subcutaneous fat). These infections typically involve the perineum, lower limbs, and torso, involving extensive soft tissue destruction along with systemic signs of sepsis. They are often associated with significant mortality and morbidity, particularly because of diagnostic and treatment delays [2, 3].

Originally described in the surgical literature by Shah et al., necrotizing fasciitis of the breast is a rare phenomenon. This entity is often misdiagnosed as an abscess, cellulitis, or inflammatory breast cancer, reflecting difficulties in recognition and leading to treatment delays [3]. Predisposing risk factors for any NSTI include diabetes mellitus, obesity, peripheral vascular disease, alcoholic liver disease, immunosuppression, and skin breaches [2, 4]. It is important to note, though, that these infections can also occur in individuals with no comorbidities or ports of entry for infection [1]. Three of our patients were immune-compromised with malignancy or chemotherapy (*n* = 2) and diabetes (*n* = 1), and one had no comorbid condition except obesity.

Breast gangrene, in particular, has been reported in trauma, thrombophlebitis, pregnancy and lactation, anticoagulation, and topical belladonna application [3, 5]. However, an extensive literature search failed to identify any published cases of NSTI superimposed on breast carcinoma. In idiopathic cases, as described by Cutter [6], the initial symptom is mastalgia, which progresses to skin induration and a peau d'orange appearance [5]. Although three of our patients were immune compromised, the inciting events of our series were ruptured breast cancer, idiopathic infected sebaceous cysts, and insect bites, respectively.

Common manifestations of NSTIs of the limbs, trunk, and perineum include skin erythema, edema, and pain out of proportion to exam findings in up to 75% of cases. Additionally, up to 50% of patients have palpable crepitus with skin bullae or necrosis [3], like ours.

NSTIs of the breast, however, can prove to be a diagnostic challenge for surgeons. An extensive blood supply to the breast contributes to a delay in skin manifestations, with most patients (including the cases presented here) presenting days to weeks after the onset of symptoms [7]. Furthermore, as demonstrated in the first case in this series, the density of breast tissue often makes it difficult to appreciate tell-tale signs like crepitus; similarly, there was no palpable crepitus noted on our third and fourth patients; however, skin bullae were noted on our fourth patient.

NSTIs are broadly categorized into four types. Type I involves mixed aerobic and anaerobic bacterial infections, often presenting as fulminant sepsis. Type II infections are generally mono-microbial (typically group A or other beta-hemolytic streptococci) and often occur in patients with significant risk factors [2, 3]. Type III (monomicrobial) infections are uncommon and caused by Gram-negative bacteria like *Clostridium*, *Klebsiella*, or *Vibrio* species. Type IV infections, on the other hand, are fungal in nature [7]. A 2020 review of twenty-five patients by Konik and Huang found that polymicrobial (type II) organisms were the most common cause of NSTIs of the breast [4]. Interestingly, two of our patients (first and last) cultures grew *E. coli* only (an uncommon mono-microbial cause); the second patient's culture placed her in the type I category, whereas the third patient grew both *E. coli* and *Klebsiella*.

In addition to clinical appearance, the presence of systemic signs of toxicity lends itself to the diagnosis of NSTIs. The Laboratory Risk Indicator for Necrotizing Fasciitis (LRINEC score) is a clinical tool that uses laboratory values and clinical signs as adjuncts in the diagnosis. Here, a score of 6 or more helps differentiate necrotizing from non-NSTIs [8]. The LRINEC score was not calculated for the cases in this series as C-reactive protein was not available at the time of the consult. However, the clinical appearance, along with systemic signs and the additional information provided by CT, was considered sufficient to make a diagnosis and a decision for management.

Various imaging modalities have been used to aid in the diagnosis of NSTIs, especially in cases where the diagnosis may not be immediately apparent. Ultrasound can detect the thickening of the deep fascia and fluid collections. CT can identify fat stranding and subcutaneous gas characteristics in these infections. The CT scan for our first and second patients revealed the presence of gas throughout the breast parenchyma, which supported the need for a mastectomy due to the extent of gas; however, in our third patient, the gas was limited to the lower inner quadrant of the breast, which supported a wide debridement instead of a mastectomy. The additional benefit of CT in our first case was that it provided some degree of the staging of her breast carcinoma and enabled a decision for radical versus modified radical mastectomy. This finding also supported the aggressive nature of her disease: progression to pectoral involvement despite multiple lines of neoadjuvant chemotherapy.

On the other hand, the question of whether a CT is necessary was raised in the management of our second case. The presence of characteristic clinical and systemic signs of NSTI, along with suspicious X-ray features, suggested that a CT was not necessary for diagnosis. No diagnostic imaging was performed when it was first described by Shah et al., likely because of the limited availability of CT at that time [9]. However, the advantage CT provided in our case was helping to determine the extent of infection and whether breast salvage was possible or not, as proven in our third case. No radiological imaging was done for our last case as our CT scan machine was temporarily down that day.

Other adjuncts, like Magnetic Resonance Imaging, have been described, especially as they provide a more detailed view of the extent of soft tissue infection. However, its specificity is low (46–86%) and, in a resource-limited setting like ours, is not readily available at times of surgical emergency [3].

A multidisciplinary approach to treating NSTIs of the breast is strongly recommended and involves input from breast surgeons, plastic surgeons, critical care specialists, microbiologists, and radiologists. This provides the platform for better patient outcomes with clear planning and decisive perioperative management [8]. Shah et al. suggested a 6-point management plan for treatment, which includes [9]: early surgical referral resuscitation, antibiotic coverage, and Intensive Care Unit referral diagnostic incision with gram stain and culture of pus radical “pseudo-tumor” excision re-exploration after twenty-four hours delayed closure several months after recovery with plastic surgery input.

Marks et al. subsequently suggested an abbreviated diagnostic and management triad based on a modification of this 6-point system [10].

Early debridement and antibiotic coverage significantly reduce the morbidity and mortality associated with NSTIs. In cases involving the breast, though, reports supported mastectomy as the most commonly performed procedure (52% of cases in two systematic reviews) [3].

The mastectomy rate in our series was 25% (*n* = 1) due to the rupture of neglected giant breast cancer, and the mortality rate was 50%; however, this was not related to the necrotizing fasciitis of the breasts. One of our patients (the first case) was diagnosed due to the advanced stage of her breast cancer (from brain metastasis) after 3–4 months of surgery, and in the third case, it was due to her pre-existing bilateral PEs after 4 weeks of surgery.

More recently, however, staged debridement has been suggested rather than immediate mastectomy as a treatment strategy [4]. Although breast salvage is possible, it is important to recognize that all necrotic tissue must be removed at the time of debridement for infection control. In severe cases, early mastectomy can be lifesaving, as was evident in the cases presented here. Even after mastectomy, it is recommended that a second look be undertaken within 24–48 hours to ensure adequate source control [4] (as in our second and third cases).

In terms of reconstruction, split-thickness skin grafts were used in most documented case reports. Reconstructive options, though, depend on the volume of residual breast tissue, the size of the open wound, the clearance of all infections, and the stability of the patient. This can be done relatively early after initial debridement, provided all of these factors are optimal.

Other potential reconstructive options include delayed primary closure (as in our first case), local flap advancement (as in our second case), local breast tissue rearrangement (an option for our third and fourth cases) with or without contralateral symmetrization (as in our fourth case), and pedicle flap reconstruction [4].

## 7. Conclusion

A prompt diagnosis of breast NSTIs is crucial for saving a life as well as potentially salvaging the breast. A diagnosis can be readily made once clear clinical signs are apparent, but when not, radiologic adjuncts can be beneficial in achieving a diagnosis and guiding surgical treatment. These patients require aggressive resuscitation and control of infection with debridement until healthy, viable tissue is encountered. This often requires a mastectomy. Re-exploration and re-debridement are necessary until all infection is cleared, and reconstruction may then be considered once it is feasible.

## Data Availability

Data supporting this research article are available from the corresponding author or the first author on reasonable request.
